# Annotations

**Published:** 1890-08-23

**Authors:** 


### Annotations.

**An Honourable Shame.**

The "spray" was the child of Sir Joseph Lister; and was at one time very dear to his heart. Like many other enthusiastic fathers, Sir Joseph used great and persuasive energy in pushing his offspring forward in life. Yet this is what he said of his darling at the International Medical Congress the other day: "As regards the spray, I feel ashamed that I should ever have recommended it for destroying the microbes of the air. If we watch the formation of the spray, and observe how its narrow initial cone expands as it advances, with fresh portions of air continually drawn into its vortex, we see that many of the microbes in it, having only just come under its influence, cannot possibly have been deprived of their vitality. Yet there was a time when I assumed that such was the case, and, trusting the spray implicitly as an atmosphere free from living organisms, omitted various precautions which I had before supposed to be essential." This passage, in which a man of the purest scientific temper apologises for having imposed a useless practice upon the world, will probably be as memorable in the history of surgery as it is honourable to its author. A great number of persons have performed every variety of surgical operation, both with and without the spray, during the last twenty years. These practical experimenters have demonstrated that, other things being equal, operations of all kinds have succeeded quite as well without the spray as with it. It may be taken as settled, therefore, that the surgical spray will now go out of use. It is of importance to mark such a fact in the history of modern surgery. But it is of a thousand times greater importance to point out to as large a number of all classes of people as possible the example of Sir Joseph Lister as a man of science. For many years Sir Joseph practised a method with the full sanction of his intellect and the highest approval of his conscience. There was a time when he would have staked his reputation on the life-saving value of his "spray." Yet now, being fully convinced that the spray has not, and cannot have, the value he attached to it, he confesses his mistake; and so far as he can makes full amends to the world. Does this mean, then, that Sir Joseph Lister's life work is a failure? In no sense whatever is it so! On the contrary, his work in the bacterial field stands unquestioned, and has received the amplest confirmation from scientists in all parts of the world. Moreover, the spray itself, though comparatively futile as a purifier, has been the pioneer of what may be called absolutely clean surgery; and in that capacity has led the most important surgical advance of modern times. Both in his successes and in his failures Sir Joseph Lister has been an example not only to the medical and scientific worlds, but to all men who have eyes to see, of that painstaking labour which is true genius, and of that candour and honesty which mark the highest scientific and moral excellence. If this peculiar and noble temper were carried into every department of human life and activity the development of man would be complete.

**Professor Huxley on Doctor's Latin.**

Professor Huxley's preference for scientific, as contrasted with classical education, has long been familiar to those who set store by his opinions. It is quite what might have been expected that he should seize upon Dr. Wade's address to the members of the British Medical Association, and use it as a hammer to drive home to the public mind one of his favourite ideas. If ever it is proper to set a scientific over against a classical education, it is particularly so in the case of medical training. A medical student must be scientifically trained, whether he is classically educated or not. Professor Huxley points out, with all the energy and directness of which he is so conspicuous a master, that eye, or ear, or limb, or even life may at any moment lie at the mercy of the first medical man we can call in. Competent knowledge and skill on the part of such a medical man may save sight or hearing or life. The want of such knowledge may insure the destruction of one and all. When, therefore, the distinguished controversialist insists upon first-hand knowledge and practical training for the medical student, no single person of any kind of education will for a moment say him nay. But this is not the whole of the question. If space permitted it could easily be shown that a classical education is of advantage to the doctor, to the scientist, and to the man. Other things being equal, the classically educated doctor, scientist, or plain man will have advantages which his confrère not so educated will not possess. In the present controversy Professor Huxley confines himself to the Latin question; and another of the correspondents of the *Times* enters a pathetic plea for the abolition, or at least for the rendering optional, of Greek. It seems to us as plain as possible that even from the standpoint of medical and scientific education alone both these controversialists are entirely wrong. For, however it has come about, the fact is that both medicine and general science are to a large extent packed and encased in Latin and Greek terminology. The student who has no knowledge of either of these tongues finds himself constantly impeded by his want of understanding of terms and their uses; and on the other hand, the student who possesses a good grammatical knowledge of both Latin and Greek finds his scientific studies greatly facilitated thereby. Of this no one who knows medical students intimately entertains the least doubt. We do not insist that medical and scientific students should be able to write Latin verses, or to construct Greek plays. That would be absurd! But we do affirm that that medical or science student who has no knowledge whatever of either of the classical tongues is like a soldier who enters into battle having left an important part of his equipment at home. The general question as to whether what is ordinarily known as a liberal education is of priceless value to doctors as well as to other men we do not now consider. But we repeat that the medical student, no more than any other man, can work with tools which he only half understands the use of. If Professor Huxley admits that formulated knowledge is necessary for the doctor as well as mere manual dexterity, then he must also admit that the means of making that knowledge clear, precise, and full, ought to be in every student's possession. A good grammatical acquaintance with both Latin and Greek is essential for the proper understanding of the medical and the general science of the times.

**Cyclists and Rupture.**

Medical opinions have been publicly expressed to the effect that cycling exposes the cyclist to the risk of rupture. The *British Medical Journal* does not believe in the danger; nor is it easy to see where the risk comes in, provided the machine be properly constructed and the rider knows how to adapt himself to it. The writer in the *British Medical* makes several criticisms which cyclists, as a class, will do well to bear in mind. Among other things, he states that hardly five per cent. of the riders make any attempt to fit themselves to their machine; that, as a rule, the handles are much too low; and that the seat is either too far back or too far forward. The conclusion is that every rider must take more pains in the selection of his machine, and if he cannot find one ready made which fulfils the conditions of having the handles at a convenient height, the feet in the right position, and the seat at the proper angle, he should either get one made to order or altered to suit his convenience and comfort. The subject is well worthy of attention, since cycling is not only a highly popular form of exercise, but can easily be made as valuable as it is popular. So important, indeed, does it appear from a health point of view, that it might very well form a leading topic for discussion at a Congress of Cyclists.

